# Examining the relationship between social determinants of health with daily tobacco use, binge-drinking, and daily cannabis use

**DOI:** 10.1371/journal.pone.0343677

**Published:** 2026-03-18

**Authors:** Zoe Lindenfeld, Ellen T. Kurtzman

**Affiliations:** Edward J. Bloustein School of Planning and Public Policy, Rutgers University, New Brunswick, New Jersey, United States of America; University of Saskatchewan, CANADA

## Abstract

**Introduction:**

There is growing recognition that social determinants of health (SDOH) shape health behaviors in powerful ways. Given that risky substance use remains a persistent public health problem, the relationship between SDOH and substance use merits investigation. The objective of this study is to examine the relationship between various social determinants of health (SDOH) with binge drinking, daily tobacco use, and daily cannabis consumption.

**Methods:**

Using the most recent Behavioral Risk Factor Surveillance System data (2022–2023) we conducted two sets of logistic regression models. The first examined relationships between our three outcomes and 13 separate SDOH measures. The second assessed a categorical, composite SDOH measure and our outcomes.

**Results:**

Frequent stress was associated with significantly higher odds of each outcome (binge drinking: AOR 1.27, 95% CI: 1.13–1.42; tobacco: AOR 1.16, 95% CI: 1.02–1.32; cannabis: AOR 1.43, 95% CI: 1.22–1.68). Having a recent medical checkup was associated with lower odds (binge drinking: AOR 0.84, 95% CI: 0.75–0.95; tobacco: AOR 0.77, 95% CI: 0.67–0.88; cannabis: AOR 0.82, 95% CI: 0.71–0.96). Higher cumulative SDOH-needs were associated with significantly higher odds. For cannabis: 1–3 needs: AOR 1.54, 95% CI: 1.27–1.87; 4–6 needs: AOR 3.35, 95% CI: 2.64–4.25; 7–13 needs: AOR 4.61, 95% CI: 3.32–6.41. Similar patterns were observed for other outcomes.

**Conclusions:**

Findings should inform interventions to reduce substance use by directly addressing SDOH associated with increased risk and by supporting the co-location of social and health services to better meet individuals’ complex needs.

## Introduction

The social determinants of health (SDOH), defined as the non-medical factors that impact health such as income, education, housing stability, and neighborhood environment, play a critical role in shaping a wide range of health behaviors. A robust body of literature demonstrates that SDOH strongly influence behaviors related to diet, physical activity, sleep, and stress management [1–3]. This is particularly relevant in the United States (US), where features of social and built environments shape outcomes in ways that differ from other high-income countries. For example, healthcare access is determined by insurance coverage and employment rather than universal provision [4], higher education is expensive and has a more significant presence of private options [5], and built environments are often low-density and car-dependent [6]. As such, in the us, SDOH have been shown to have a strong influence on health outcomes and behaviors [7,8] and highlight how social context can constrain or enable individuals’ abilities to engage in health-promoting behaviors. An advantage of using SDOH to identify disparities in health outcomes is that these factors are more modifiable than demographic or genetic characteristics and can be addressed through targeted policies and social/public health interventions (e.g., food banks, support networks.) Given that substance use, including risky use of alcohol, cannabis and tobacco, remain a persistent public health problem, the relationship between SDOH and substance use merits rigorous investigation [9,10].

Prior studies have examined the relationship between selected SDOH and patterns of tobacco, cannabis, and alcohol consumption, finding mixed results [11]. For example, studies have found that rates of risky alcohol use are higher among individuals who are unemployed [12], less educated [13], reside in rural areas [14], have low incomes [12], or are members of racial or ethnic minority groups [12,15]. Other studies have found higher rates of risky alcohol use among individuals who are White, educated, and have higher income levels [16–18]. The findings with regard to tobacco and cannabis are less varied, with studies finding higher rates of tobacco use among individuals with low incomes, criminal-justice involvement, and who live in rural communities [19], and higher rates of cannabis use among individuals who have lower levels of education attainment, lower incomes [20,21], and experience housing insecurity [21].

Mixed findings related to risky alcohol use may be attributed to differences in study populations, including research conducted outside the US [11–13], or those set in a single state [17,18]. Additionally, studies on alcohol, tobacco, and cannabis use have often relied on limited or imprecise measures of SDOH, such as using low-income as a vague proxy for low SES [19], and used varied measures of alcohol and tobacco consumption [22]. Overall, there is a need for research examining the relationship between social determinants of health and risky tobacco use, cannabis use, and alcohol use using nationally representative US samples, consistent measures of alcohol, tobacco, and cannabis use, and more comprehensive, multidimensional measures of SDOH.

In this study, we address this gap in knowledge by using data from the 2022–2023 Behavioral Risk Factor Surveillance System (BRFSS), an ongoing, US-based health-related survey worldwide [23], to examine the association between multiple measures of SDOH with binge drinking, daily tobacco use, and daily cannabis use. We focus on tobacco, alcohol, and cannabis because they are the most commonly used substances in US and are legal for adult use in many states [10], making them particularly relevant for population-level public health research. Use of these substances occurs within a heterogeneous and evolving policy context, particularly for cannabis and tobacco, where state and local policies related to legalization, decriminalization [24], product availability, taxation, and regulation (e.g., flavored products and e-cigarettes) vary widely [25] and may shape patterns of use. The inclusion of the new SDOH module within the BRFSS, which was introduced in 2022, provides a unique opportunity to examine how social and structural factors shape risky substance use patterns at a population level. This rich, representative dataset enables a nuanced analysis that can advance our understanding of the pathways linking SDOH and substance use. Specifically, this analysis examines how distinct domains of SDOH, including economic insecurity, housing instability, access to care, and social support, are differentially associated with binge drinking, daily tobacco use, and daily cannabis use, allowing for a more granular assessment that is often obscured in analyses using a single measure or less specific measures of SDOH.

## Materials and methods

### Data source

We analyzed 2022–2023 data from the BRFSS, a nationally representative, cross-sectional survey of US adults administered by the Centers for Disease Control and Prevention (CDC) and US state health departments using landline and cellular interviews. BRFSS uses a multistage cluster design to survey non-institutionalized adults aged 18 years and older on health status, behaviors, chronic conditions, and preventive service use. More than 400,000 surveys are conducted annually, with response rates of 45.0% (2022) and 44.7% (2023) [23].

Tobacco and alcohol use measures are part of the BRFSS core questionnaire. Cannabis use (Marijuana Use module) and social determinants of health (Social Determinants and Health Equity module) are optional [26]. The Cannabis Use module was implemented by 24 US states in 2022 and 19 US states in 2023; the SDOH module was implemented by 52 US states in 2022 and 41 in 2023 (S1 Table in the Supplemental Information).

### Primary outcomes

We examined three past-month substance use outcomes: (1) binge drinking, defined as one or more episodes of binge drinking in the past 30 days (5 or more drinks on one occasion for men or 4 or more drinks on one occasion for women) [27]; (2) daily tobacco use, defined as reporting cigarette or e-cigarette use every day; and (3) daily cannabis use, defined as cannabis use on 20 or more days in the past month [28]. We use daily consumption of tobacco and cannabis as measures of risk consumption given that these are considered excessive and harmful levels of consumption in the literature, and are associated with increased risk of health complications and dependence [29,30]. Similarly, past-month binge drinking is a validated measure of risky alcohol consumption, and is associated with increased risk of impairment, unsafe behavior, development of chronic health conditions, and mortality [31]. All variables were coded dichotomously (1 = meets criteria; 0 = otherwise). A full list of BRFSS variables is provided in S2 Table in the Supplemental Information.

### SDOH conceptual framework and selection of independent variables

We selected 13 SDOH indicators guided by the CMS Accountable Health Communities Health-Related Social Needs Screening Tool [32,33]. BRFSS items were mapped to AHC domains (housing, food insecurity, transportation, utilities, interpersonal safety) and supplementary domains (financial strain, employment, education, social support, physical activity, mental health, disability). The variable selection process is documented in Table 1. Although several SDOH variables in this analysis are single-item measures, prior research using BRFSS data has demonstrated that these items exhibit theoretically consistent associations with relevant health outcomes, supporting their validity for examining SDOH at the population level [34–36]. Additionally, drawing on an approach to Adverse Childhood Experiences (ACEs) composite measure development described in the literature [37], we also created an SDOH categorical cumulative risk score (“SDOH score”). The SDOH score reflected the total number of SDOH exposures for each respondent, reflecting SDOH burden, categorized as follows: 0 SDOH, 1–3 SDOH, 4–6 SDOH, and 7–13 SDOH.

**Table 1:** Selection of Social Determinant of Health Variables Included in Main Models.

Domain	AHS Question(s)	BRFSS Question	Notes
**Housing instability**	What is your living situation today? Think about the place you live. Do you have problems with any of the following?	During the last 12 months, was there a time when you were not able to pay your mortgage, rent or utility bills?^**a b**^	Included in models predicting all three study outcomes
**Food insecurity**	Within the past 12 months, you worried that your food would run out before you got money to buy more. Within the past 12 months, the food you bought just didn’t last and you didn’t have money to get more.	1. During the past 12 months how often did the food that you bought not last, and you didn’t have money to get more?^**a b**^ 2. During the past 12 months, have you received food stamps, also called SNAP, the Supplemental Nutrition Assistance Program on an EBT card?^**a b**^	Included in models predicting all three study outcomes
**Transportation problems**	In the past 12 months, has lack of reliable transportation kept you from medical appointments, meetings, work or from getting things needed for daily living?	During the past 12 months has a lack of reliable transportation kept you from medical appointments, meetings, work, or from getting things needed for daily living?^**a b**^	Included in models predicting all three study outcomes
**Utility help needs**	In the past 12 months has the electric, gas, oil, or water company threatened to shut off services in your home?	During the last 12 months was there a time when an electric, gas, oil, or water company threatened to shut off services?^**a b**^	Included in models predicting all three study outcomes
**Interpersonal safety**	How often does anyone, including family and friends, physically hurt you? How often does anyone, including family and friends, insult or talk down to you? How often does anyone, including family and friends, threaten you with harm? How often does anyone, including family and friends, scream or curse at you?	How often do you get the social and emotional support that you need?^**a b**^	Included in models predicting all three study outcomes
**Financial strain**	How hard is it for you to pay for the very basics like food, housing, medical care, and heating?	Was there a time in the past 12 months when you needed to see a doctor but could not because you could not afford it?^**b**^	Included in models predicting all three study outcomes
**Employment**	Do you want help finding or keeping work or a job?	In the past 12 months have you lost employment or had hours reduced?^**a b**^	Included in models predicting all three study outcomes
**Family and community support**	If for any reason you need help with day-to-day activities such as bathing, preparing meals, shopping, managing finances, etc., do you get the help you need? How often do you feel lonely or isolated from those around you?	How often do you feel lonely?^**a b**^	*Excluded due to high missingness*
**Education**	Do you speak a language other than English at home? Do you want help with school or training? For example, starting or completing job training or getting a high school diploma, GED or equivalent.	What is the highest grade or year of school you completed?	*Included as demographic variable*
**Physical activity**	In the last 30 days, other than the activities you did for work, on average, how many days per week did you engage in moderate exercise (like walking fast, running, jogging, dancing, swimming, biking, or other similar activities)? On average, how many minutes did you usually spend exercising at this level on one of those days?	During the past month, other than your regular job, did you participate in any physical activities or exercises such as running, calisthenics, golf, gardening, or walking for exercise? ^**b**^	Included in models predicting all three study outcomes
**Substance use**	How many times in the past 12 months have you had 5 or more drinks in a day (males) or 4 or more drinks in a day (females)? One drink is 12 ounces of beer, 5 ounces of wine, or 1.5 ounces of 80-proof spirits. How many times in the past 12 months have you used tobacco products (like cigarettes, cigars, snuff, chew, electronic cigarettes)?	Considering all types of alcoholic beverages, how many times during the past 30 days did you have 5 or more drinks for men or 4 or more drinks for women on an occasion? During the past 30 days, on how many days did you use marijuana or cannabis? Do you now smoke cigarettes every day, some days, or not at all?	*Included as model outcomes*
**Mental health**	Over the past 2 weeks, how often have you been bothered by any of the following problems? a. Little interest or pleasure in doing things? b. Feeling down, depressed, or hopeless? Stress means a situation in which a person feels tense, restless, nervous, or anxious, or is unable to sleep at night because his or her mind is troubled all the time. Do you feel this kind of stress these days?	Stress means a situation in which a person feels tense, restless, nervous or anxious or is unable to sleep at night because their mind is troubled all the time. Within the last 30 days, how often have you felt this kind of stress?^**a b**^	Included in models predicting all three study outcomes
**Disabilities**	Because of a physical, mental, or emotional condition, do you have serious difficulty concentrating, remembering, or making decisions? Because of a physical, mental, or emotional condition, do you have difficulty doing errands alone such as visiting a doctor’s office or shopping?	1. Because of a physical, mental, or emotional condition, do you have serious difficulty concentrating, remembering, or making decisions? ^**b**^ 2. Because of a physical, mental, or emotional condition, do you have difficulty doing errands alone such as visiting a doctor’s office or shopping? ^**b**^	Included in models predicting all three study outcomes

^a^Variables drawn from the BRFSS Social Determinants and Health Equity module.

^b^Included in constructed SDOH composite variable.

### Demographic covariates

We included a number of covariates derived from the BRFSS, including demographic characteristics (age, race/ethnicity, marital status, veteran status, employment status) and healthcare access variables (insurance type and whether the respondent had a recent routine checkup).

### Analytic sample

Data were restricted to US states implementing both the Cannabis Use and SDOH modules in the same year. The final analytic sample included 149,021 adults from 14 US states across 2022–2023 (S3 Table in the Supplemental Information). Respondents missing data on a given substance use outcome were not included in that specific analysis; no imputation was performed, and they remained in the dataset for other analyses [38].

### Statistical analysis

We calculated descriptive statistics for the full sample and by each outcome group (binge drinking, daily tobacco use, daily cannabis use), reporting weighted percentages and 95% confidence intervals. We then fit three separate logistic regression models for each outcome. We limited the covariates in the first model for each outcome to only the demographic covariates. The second model included both the demographic covariates as well as the 13, dichotomous SDOH variables; this allowed us to estimate “the impact of the occurrence of each specific SDOH exposure (given the presence/absence of the other SDOH exposures),” [37] which is expected to function well for events which are highly correlated, such as SDOH capturing varying measures of poverty. The third model included the demographic variables as well as the SDOH categorical cumulative risk score.

Models for daily cannabis use additionally adjusted for US state cannabis legalization status. Legalization status by state-year was determined from publicly available sources and coded as legal vs. not legal (S4 Table in the Supplemental Information). Variance inflation factors (VIFs) were assessed; education showed higher VIF in one model but was retained for conceptual reasons. All analyses applied BRFSS survey weights and accounted for complex design using Stata SE 18. The study was deemed non–human subjects research by Rutgers IRB. This study followed the STROBE (Strengthening the Reporting of Observational Studies in Epidemiology) guidelines.

## Results

There were 149,021 observations in our analytic sample. Of these, 45.07% (n = 67,169) responded to survey questions related to daily tobacco use, 48.16% (n = 71,781) responded to questions related to binge drinking, and 84.59% (n = 126,058) responded to questions focused on daily cannabis use. These observations are not mutually exclusive.

Among respondents to questions related to substance use, 32.33% (n = 15,372) reported daily tobacco use, 30.01% (n = 18,087) reported binge drinking, and 7.34% (n = 7,890) reported daily cannabis use.

### Descriptive statistics- Unadjusted prevalence of demographic and SDOH variables

Table 2 presents descriptive statistics, including demographic characteristics, for all respondents and for those reporting daily tobacco use, daily cannabis use, and binge drinking. Regarding SDOH burden, 29.09% (95% CI: 28.60–29.59) of respondents reported zero SDOH, 55.37% (95% CI: 54.81–55.92) reported 1–3 SDOH, 12.64% (95% CI: 12.26–13.03) reported 4–6 SDOH, and 2.89% (95% CI: 2.68–3.11) reported 7–13 SDOH. Higher SDOH burden was more common among substance users: 7.66% (95% CI: 6.85–8.57) of daily tobacco users, 8.79% (95% CI: 7.65–10.08) of daily cannabis users, and 4.61% (95% CI: 4.08–5.22) of binge drinkers reported 7–13 SDOH. Most respondents (77.05%; 95% CI: 76.64–77.46) reported a medical check-up in the past year, compared to 62.23% (95% CI: 60.11–64.31) of daily tobacco users, 68.17% (95% CI: 66.32–69.96) of daily cannabis users, and 72.18% (95% CI: 70.93–73.40) of binge drinkers. Indicators of economic insecurity were also more common among substance use groups. For example, inability to afford medical care was reported by 10.34% (95% CI: 10.03–10.66) of the full sample versus 21.13% (95% CI: 19.44–22.91) of daily cannabis users, 19.52% (95% CI: 17.88–21.27) of daily tobacco users, and 13.09% (95% CI: 12.09–14.16) of binge drinkers.

**Table 2:** Descriptive statistics of respondents, overall and among those reporting daily tobacco use, daily cannabis use, and binge drinking behaviors*.

Variable	All Respondents (n = 149, 021) % (95% CI)	Respondents Reporting Tobacco Use (n = 15,372) % (95% CI)	Respondents Reporting Daily Cannabis Use (n = 7,890) % (95% CI)	Respondents Reporting Binge Drinking (n = 18,087) % (95% CI)
**Age**				
*18-24*	12.07 (11.71-12.45)	12.48 (11.35-13.70)	19.18 (17.34-21.17)	16.95 (15.89-18.06)
*25-34*	15.90 (15.52-16.29)	18.51 (17.31-19.78)	25.27 (23.59-27.02)	23.90 (22.81-25.02)
*35-44*	16.01 (15.66-16.37)	20.38 (19.28-21.53)	21.42 (19.87-23.05)	21.48 (20.43-22.57)
*45-54*	15.16 (14.82-15.50)	15.46 (14.49-16.48)	13.32 (11.98-14.78)	16.48 (15.56-17.44)
*55-64*	16.53 (16.19-16.88)	19.00 (17.83-20.23)	11.65 (10.61-12.79)	12.84 (12.07-13.65)
*65+*	24.29 (23.91-24.68)	14.14 (13.31-15.01)	9.13 (8.14-10.22)	8.32 (7.72-8.95)
**Race**				
*White*	71.08 (70.42-71.72)	76.79 (75.08-78.41)	68.02 (65.61-70.33)	74.51 (72.92-76.04)
*Black/African American*	10.67 (10.25-11.11)	8.45 (7.42-9.62)	13.75 (12.06-15.63)	8.42 (7.47-8.47)
*Hispanic/Latino*	10.14 (9.70-10.60)	7.21 (6.29-8.25)	9.62 (8.15-11.32)	10.13 (9.11-11.25)
*Asian*	2.88 (2.57-3.22)	1.70 (1.07-2.68)	0.72 (0.39-1.30)	2.22 (1.63-3.01)
*Native American/AIAN*	1.62 (1.46-1.81)	2.04 (1.61-2.59)	2.41 (1.82-3.18)	1.35 (2.04-1.76)
*Other*	3.58 (3.30-3.89)	3.78 (3.09-4.61)	5.46 (4.26-6.97)	3.33 (2.64-4.20)
**Sex**				
*Female*	51.37 (50.89-51.86)	47.99 (46.50-49.47)	42.27 (40.26-44.30)	38.89 (37.66-40.13)
*Male*	48.62 (48.13-49.10)	52.00 (50.51-53.49)	57.72 (55.69-59.73)	61.20 (59.86-62.33)
**Married**	49.97 (49.48-50.45)	35.52 (34.14-36.91)	31.76 (29.97-33.60)	45.85 (44.56-47.16)
**Veteran**	10.31 (10.04-10.59)	10.92 (10.01-11.90)	8.41 (7.47-9.47)	9.30 (8.61-10.04)
**Health Insurance Type**				
*Private/Employer*	49.04 (048.46-49.61)	41.07 (39.44-42.72)	41.04 (38.60-43.53)	61.89 (60.45-63.31)
*Medicare*	22.73 (22.28-23.18)	17.30 (16.20-18.45)	14.01 (12.61-15.54)	10.20 (9.33-10.92)
*Medicaid/CHIP*	8.24 (0.79-8.5)	15.09 (13.93-16.33)	17.26 (15.29-19.44)	6.91 (6.16-7.74)
*Other*	12.68 (12.29-13.07)	15.27 (14.18-16.43)	16.50 (14.73-18.43)	12.39 (11.45-13.40)
*Uninsured*	7.29 (6.96-7.63)	11.24 (10.17-12.41)	11.16 (9.66-12.87)	8.68 (7.81-9.64)
**Employed**	57.48 (56.99-57.97)	59.14 (57.64-60.63)	64.03 (62.04-65.97)	74.13 (72.96-75.26)
**Education**				
*Less than High School*	10.41 (10.08-10.78)	16.81 (15.62-18.06)	12.64 (11.12-14.34)	8.35 (7.40-9.29)
*High school*	28.95 (28.51-29.39)	39.16 (37.72-40.61)	35.05 (33.10-37.04)	27.99 (26.83-29.19)
*Some college*	30.32 (29.87-30.78)	32.78 (31.39-34.19)	34.53 (32.60-36.51)	32.41 (31.16-33.68)
*College graduate*	30.28 (39.89-30.69)	11.24 (10.54-11.98)	17.77 (16.54-19.07)	31.23 (30.18-32.30)
**Cumulative number of SDOH reported**				
*0*	29.09 (28.60-29.59)	14.43 (13.23-15.71)	13.36 (12.10-14.73)	24.82 (23.62-26.06)
*1-3*	55.37 (54.81-55.92)	53.69 (52.03-55.34)	51.21 (49.07-53.35)	57.72 (56.30-59.13)
*4-6*	12.64 (2.26-13.03)	24.20 (22.86-25.59)	26.61 (24.75-28.57)	14.01 (13.03-15.06)
*7-13*	2.89 (2.68-3.11)	7.66 (6.85-8.57)	8.79 (7.65-10.08)	3.43 (2.94-3.99)
**Check-up within past year**	77.05 (76.64-77.46)	68.41 (67.07-69.73)	65.40 (63.44-67.32)	67.95 (66.74-69.14)
**Cannot afford medical care**	10.34 (10.03-10.66)	17.62 (16.46-18.84)	21.13 (19.44-22.91)	12.76 (11.92-13.65)
**Receives food stamps**	10.78 (10.42-11.15)	20.75 (19.51-22.06)	20.17 (18.51-21.95)	8.36 (7.63-9.15)
**Food purchased does not last**	13.79 (13.39-14.19)	25.17 (23.78-26.62)	25.35 (23.58-27.21)	12.87 (11.97-13.83)
**Lack transportation access**	7.55 (7.24-7.87)	14.51 (13.30-15.81)	17.22 (15.68-18.87)	8.10 (7.33-8.94)
**Housing instability**	11.47 (11.09-11.85)	22.66 (21.23-24.16)	22.94 (21.21-24.78)	13.10 (12.14-14.13)
**Utility bill needs**	7.64 (7.33-7.96)	16.20 (14.93-17.56)	17.19 (15.66-18.83)	8.91 (8.12-9.77)
**Has emotional support**	91.07 (90.73-91.41)	85.39 (84.12-86.58)	83.27 (81.58-84.83)	90.18 (89.21-91.06)
**Job loss within past year**	11.94 (11.54-12.34)	18.64 (17.28-20.08)	22.10 (20.31-24.00)	15.21 (14.13-16.36)
**Exercise in past 30 days**	75.75 (75.33-76.17)	66.04 (64.60-67.46)	77.42 (75.65-79.10)	81.85 (80.82-82.85)
**Frequent stress**	38.15 (37.61-38.69)	53.22 (51.57-54.86)	56.37 (54.25-58.47)	45.80 (44.40-47.21)
**Difficulty concentrating due to disability**	13.34 (13.00-13.68)	24.90 (23.64-26.21)	29.36 (27.47-31.33)	15.00 (14.08-15.97)
**Difficulty doing errands alone due to disability**	7.86 (7.57-8.15)	14.55 (13.37-15.82)	15.27 (13.89-16.77)	6.01 (5.37-6.72)
**Resides in a state in which adult use cannabis is legal**	61.63 (6.11-6.20)	54.73 (53.32-56.14)	68.82 (67.12-70.47)	62.00 (60.85-63.14)

*95% CI – 95% confidence interval.

Source: Behavior Risk Factor Surveillance System 2022–2023.

All estimates are adjusted for sampling weight and BRFSS’ complex survey design; confidence intervals are based on standard errors computed using the linearized (or robust) variance estimator.

Frequent stress was reported by 38.15% (95% CI: 37.61–38.69) of the full sample, compared with 56.37% (95% CI: 54.25–58.47) among daily cannabis users, 53.09% (95% CI: 51.00–55.16) among daily tobacco users, and 48.61% (95% CI: 47.15–50.08) among binge drinkers. Difficulty concentrating due to a disability was reported by 13.34% (95% CI: 13.00–13.68) overall and was highest among daily cannabis users (29.36% [95% CI: 27.47–31.33]) and daily tobacco users (27.42% [95% CI: 25.59–29.34]) compared to binge drinkers (17.65% [95% CI: 16.54–18.81]). Finally, 61.63% (95% CI: 61.11–62.20) of respondents resided in a US state where adult-use cannabis was legal, compared to 68.82% (95% CI: 67.12–70.47) of daily cannabis users, 64.15% (95% CI: 62.00–66.24) of daily tobacco users, and 65.73% (95% CI: 64.39–67.06) of binge drinkers.

### Logistic regression models with demographic variables and a categorical SDOH composite variable predicting daily cannabis use, binge drinking, and daily tobacco use

Results from the models with demographic variables and a categorical SDOH composite variable as the primary predictor can be found in Table 3. Having more SDOH-related needs compared to zero SDOH-needs was associated with significantly and increasingly higher odds of reporting risky substance use: daily cannabis use (1–3 needs: AOR- 1.54, 95% CI: 1.27–1.87; 4–6 needs: AOR-3.35, 95% CI: 2.64–4.25; 7–13 needs: AOR- 4.61, 95% CI: 3.32–6.41), binge drinking (1–3 needs: AOR- 1.33, 95% CI: 1.19–1.49; 4–6 needs: AOR- 1.73, 95% CI: 1.41–2.13; 7–13 needs: AOR- 1.62, 95% CI: 1.14–2.30), and daily tobacco use (1–3 needs: AOR- 1.58, 95% CI: 1.35–1.84; 4–6 needs: AOR- 2.20, 95% CI: 1.80–2.71; 7–13 needs: AOR- 2.56, 95% CI: 1.88–3.48). Results from the models with only demographic variables as predictors can be found in S5 Table in the Supplemental Information.

**Table 3:** Logistic regression models with cumulative number of SDOH predicting daily tobacco use, daily cannabis use, and binge drinking behaviors, Adjusted Odds Ratios and 95% Confidence Intervals.

Variable	Daily Cannabis (n = 38,386)	Binge Drinking (n = 20,741)	Daily Tobacco (n = 16,914)
**Age (based category: 18–24)**			
*25-34*	1.03	0.92	0.50**
	(0.81 - 1.31)	(0.73 - 1.14)	(0.36 - 0.70)
*35-44*	0.95	1.04	0.50**
	(0.74 - 1.22)	(0.83 - 1.29)	(0.36 - 0.69)
*45-54*	0.54**	0.88	0.45**
	(0.42 - 0.70)	(0.70 - 1.09)	(0.33 - 0.62)
*55-64*	0.47**	0.54**	0.37**
	(0.37 - 0.60)	(0.43 - 0.67)	(0.27 - 0.51)
*65+*	0.22**	0.34**	0.18**
	(0.16 - 0.30)	(0.26 - 0.45)	(0.13 - 0.25)
**Race (base category: White)**			
*Black/African American*	1.17	0.67**	0.72*
	(0.89 - 1.54)	(0.51 - 0.87)	(0.54 - 0.97)
*Hispanic/Latino*	0.45**	0.86	0.51**
	(0.34 - 0.59)	(0.70 - 1.07)	(0.40 - 0.65)
*Asian*	0.24**	0.85	1.92
	(0.11 - 0.56)	(0.48 - 1.51)	(0.71 - 5.17)
*Native American/AIAN*	0.75	1.3	0.54**
	(0.53 - 1.06)	(0.86 - 1.97)	(0.37 - 0.78)
*Other*	1.24	0.91	0.76
	(0.80 - 1.92)	(0.60 - 1.38)	(0.50 - 1.13)
**Sex (based category: female)**			
*Male*	1.74**	1.71**	0.86*
	(1.51 - 2.01)	(1.54 - 1.90)	(0.76 - 0.98)
**Married**	0.70**	0.74**	0.67**
	(0.60 - 0.83)	(0.66 - 0.83)	(0.59 - 0.75)
**Veteran**	1.08	0.83*	1.08
	(0.86 - 1.35)	(0.70 - 0.99)	(0.90 - 1.30)
**Health Insurance Type (based category: Private/Employer)**			
*Medicare*	1.49**	0.91	1.13
	(1.14 - 1.95)	(0.73 - 1.13)	(0.92 - 1.37)
*Medicaid/CHIP*	1.77**	0.86	1.67**
	(1.39 - 2.25)	(0.68 - 1.09)	(1.35 - 2.07)
*Other*	1.28*	0.96	1.14
	(1.03 - 1.60)	(0.80 - 1.15)	(0.95 - 1.37)
*Uninsured*	0.86	0.99	1.27
	(0.66 - 1.14)	(0.78 - 1.25)	(0.99 - 1.62)
**Employed**	1.08	1.29**	1.13
	(0.91 - 1.28)	(1.13 - 1.49)	(0.98 - 1.32)
**Education (base category: less than high school)**			
*High school*	0.87	0.78	0.86
	(0.67 - 1.14)	(0.58 - 1.04)	(0.70 - 1.06)
*some college*	0.89	0.75*	0.62**
	(0.68 - 1.16)	(0.56 - 1.00)	(0.50 - 0.77)
*College graduate*	0.64**	0.59**	0.40**
	(0.48 - 0.85)	(0.44 - 0.79)	(0.31 - 0.50)
**Resides in a state in which adult use cannabis is legal**	2.31**	---	---
	(2.02 - 2.65)	---	---
**Cumulative number of SDOH reported (base category: 0)**			
*1-3*	1.54**	1.33**	1.58**
	(1.27 - 1.87)	(1.19 - 1.49)	(1.35 - 1.84)
*4-6*	3.35**	1.73**	2.20**
	(2.64 - 4.25)	(1.41 - 2.13)	(1.80 - 2.71)
*7-13*	4.61**	1.62**	2.56**
	(3.32 - 6.41)	(1.14 - 2.30)	(1.88 - 3.48)

Source: Behavior Risk Factor Surveillance System 2022–2023.

All estimates are adjusted for sampling weight and BRFSS’ complex survey design; confidence intervals are based on standard errors computed using the linearized (or robust) variance estimator.

### Logistic regression models with demographic variables and individual SDOH variables predicting daily cannabis use, binge drinking, and daily tobacco use

**Fig 1:**
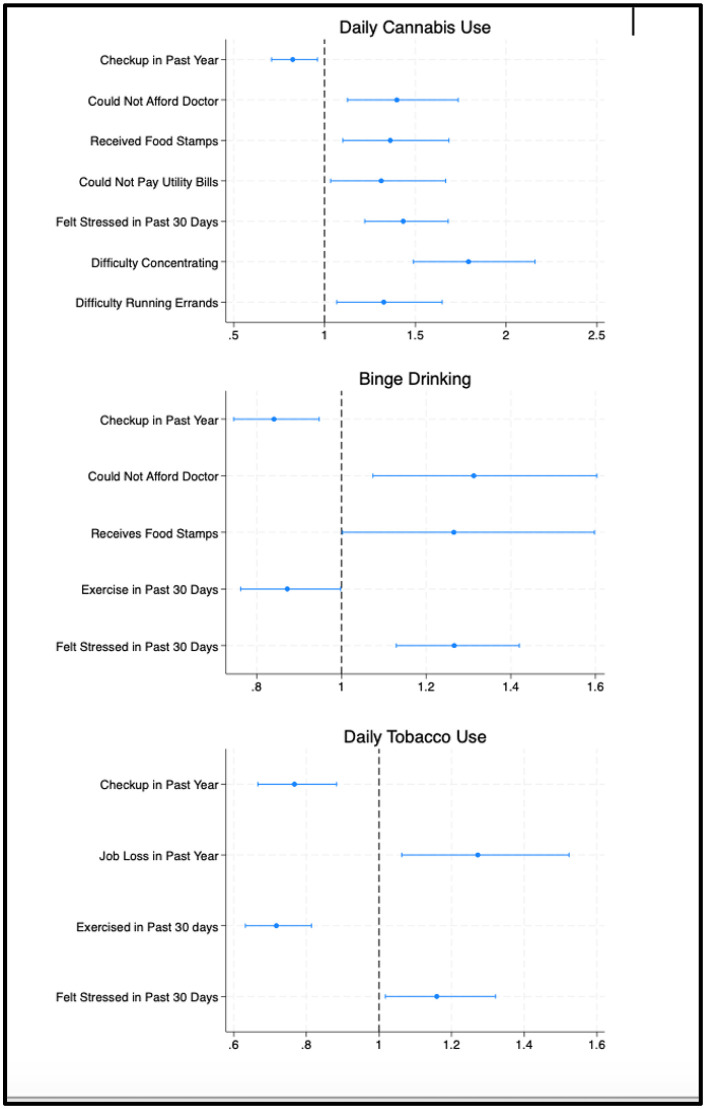
Significant SDOH predictors of daily cannabis use, binge drinking, and daily tobacco use, Adjusted Odds Ratios and 95% Confidence Intervals (n = 38,332). LEGEND: The full regression model, including the odds ratios for demographic variables and SDOH variables with p < 0.05 is reported in S6 Table in the Supplemental Information..

In the adjusted logistic regression model predicting daily cannabis use (Fig 1 and S6 Table in the Supplemental Information), several individual SDOH factors were associated with higher odds of daily use. Specifically, being unable to afford medical care (AOR: 1.50; 95% CI: 1.13–1.74), receiving food stamps (AOR: 1.36; 95% CI: 1.10–1.68), being unable to pay utility bills (AOR: 1.31; 95% CI: 1.03–1.67), feeling frequently stressed in the past 30 days (AOR: 1.43; 95% CI: 1.22–1.68), having difficulty concentrating due to a disability (AOR: 1.79; 95% CI: 1.49–2.16), and experiencing difficulty running errands alone due to a disability (AOR: 1.33; 95% CI: 1.07–1.65) were associated with significantly higher odds of daily cannabis use. Living in a US state where adult-use cannabis was legal was also strongly associated with daily use (AOR: 2.29; 95% CI: 2.00–2.63). In contrast, receiving a medical check-up in the past year was associated with lower odds of daily cannabis use (AOR: 0.82; 95% CI: 0.71–0.96).

For binge drinking, being unable to afford medical care (AOR: 1.31; 95% CI: 1.07–1.60), receiving food stamps (AOR: 1.27; 95% CI: 1.00–1.60), and feeling frequently stressed (AOR: 1.27; 95% CI: 1.13–1.42) were associated with higher odds, whereas receiving a medical check-up (AOR: 0.84; 95% CI: 0.75–0.95) and engaging in exercise (AOR: 0.87; 95% CI: 0.76–1.00) were associated with lower odds (Fig 1 and S6 Table in the Supplemental Information).

For daily tobacco use, feeling frequently stressed (AOR: 1.16; 95% CI: 1.02–1.32) and losing employment in the past year (AOR: 1.27; 95% CI: 1.06–1.52) were associated with higher odds, while receiving a medical check-up (AOR: 0.77; 95% CI: 0.67–0.88) and engaging in exercise (AOR: 0.72; 95% CI: 0.62–0.81) were associated with lower odds (Fig 1 and S6 Table in the Supplemental Information).

For each model, non-significant SDOH variables and both significant and non-significant demographic predictors are reported in S6 Table in the Supplemental Information. VIFs for each model are reported in S7 Table in the Supplemental Information.

## Discussion

This study used the most recent two years of BRFSS data to examine the relationship between various measures of SDOH and daily cannabis use, binge drinking, and daily tobacco use. These analyses use 2022–2023 data, providing insight into substance use behaviors in a post-COVID-19 context, when social, economic, and healthcare patterns may have shifted at the population level. Across all three study outcomes, having a higher number of SDOH was associated with an increased risk of each study outcome, highlighting the role of SDOH in shaping patterns of substance use. In models examining the contribution of specific SDOH, markers of stress and financial hardship were associated with reporting each outcome, whereas access to medical care and opportunities for physical activity were protective across all outcomes.

Specifically, experiencing frequent stress in the past 30 days was associated with significantly higher odds of reporting each measure of risky substance use. This is aligned with previous literature documenting how high levels of stress influence engagement in health-compromising behaviors (e.g., self harm, disordered eating, aggressive behavior,) and is highly comorbid with substance use [39]. Prior research has also examined the mechanisms underlying the relationship between frequent stress and substance use, and suggests that emotional dysregulation, or the inability to control intense emotions such as stress, plays a key role [40]. However our finding that an inability to afford medical care, job loss in the past year, being on food stamps, and an inability to pay utility bills were associated with a higher likelihood of substance use in different models, could suggest that frequent stress stemming from an inability to afford basic needs may underlie the relationship between stress and substance use. This, too, is aligned with previous literature highlighting the relationship between poverty and both stress and substance use [41]. This study confirms and extends these previous findings by including more specific measures of financial precarity that also suggest potential points of intervention.

These results are particularly salient given recent policies that require SDOH screening in medical settings. In recognition of the link between SDOH and health outcomes, key regulatory and standard-setting agencies have introduced measures and policies to gather information on patients’ SDOH. For example, the 10th revision of the International Classification of Diseases (ICD-10) included a new set of “Z-codes” to classify and document SDOH across nine categories [42]. Furthermore, in 2024, the Centers for Medicare and Medicaid Services (CMS) mandated SDOH screening across five domains during adult hospitalizations [43]. In conjunction with these new requirements, there are ongoing conversations among policymakers, healthcare administrators, and physicians surrounding how healthcare systems should address patients’ SDOH needs identified through screening [44,45]. Potential interventions include referrals to community organizations (for example, food banks or organizations that provide assistance with Medicaid enrollment) and collocating social workers or local social service resources within medical settings [46,47]; however, health care systems, particularly those serving populations with high rates of SDOH and substance use, may be hesitant and/or least able to implement these programs due to resource and bandwidth constraints [47]. By documenting the link between modifiable SDOH and high-cost behaviors such as daily tobacco use and binge drinking, which cost the US health system approximately $240 billion and $28 billion annually, respectively [48,49], our study provides additional support for SDOH programs that directly address these risk factors.

The analysis has several limitations. Most significantly, BRFSS data are self-reported, which can result in favorability bias and potentially lead to underreporting of substance use behaviors, resulting in conservative estimates of prevalence. Second, among respondents reporting substance use, the survey does not ask about their reason(s) for use nor the consequences of their use, which could contribute to confounding. Additionally, because of the wording of questions about SDOH, it is impossible to determine whether these contributed to—or resulted from— reported substance use (i.e., reverse causality). The BRFSS also does not ask respondents about their use of illicit substances such as heroin, fentanyl, or methamphetamine, which have been significant public health issues in the US and are important areas for future research regarding the SDOH-substance use relationship. The analysis does not account for family caregiving responsibilities, which are relevant factors that may influence substance use behaviors. The absence of these measures in the BRFSS limits our ability to assess how caregiver burden may modify the observed associations. Another limitation is the lack of detailed spatial data on environmental exposures and neighborhood characteristics. For example, because we did not have sub-state geographic indicators for survey respondents, measures such as ambient noise, greenness, or the density and type of local alcohol or convenience retailers were not captured, which may influence substance use behaviors and limit the ability to fully account for area-level SDOH influences. Finally, BRFSS data are cross-sectional and not longitudinal, which prevents us from making any causal claims about the relationships measured in this analysis.

### Conclusions

This study makes an important contribution to the literature on SDOH and substance use by using a large, ongoing health-related survey to demonstrate the relationship between SDOH such as food insecurity, healthcare unaffordability and frequent stress with several forms of risky substance use. Findings from this study can support healthcare practitioners, public health professionals, and policymakers in reducing rates of binge drinking, daily cannabis use, and daily tobacco use by addressing key SDOH associated with elevated risk, and by promoting protective factors, such as physical exercise, through targeted interventions and supportive policies. Co-location of social and health services may be a critical intervention for mitigating substance use risk by reducing barriers to care and addressing unmet social needs alongside treatment for substance use. Future studies should employ causal designs and assess whether interventions targeting the SDOH identified in this study reduce rates of substance use as well as morbidity and mortality attributable to substance use. Future research should also examine how specific types of employment influence substance use, as certain work environments may be associated with higher risks.

## Supporting information

S1 Table List of States that Elected to Use Cannabis and SDOH Modules by Year. Source: Behavior Risk Factor Surveillance System 2022–2023.
(DOCX)

S2 Table BRFSS Variables Used in Study. (DOCX)

S3 Table Number of Observations in the Analytic Sample by State-Year. Source: Behavior Risk Factor Surveillance System 2022–2023.
(DOCX)

S4 Table Adult Use Cannabis Legalization by State-Year. (DOCX)

S5 Table Logistic regression models with only demographic variables predicting daily tobacco use, daily cannabis use, and binge drinking behaviors, Adjusted Odds Ratios and 95% Confidence Intervals. *p < 0.05. **p < 0.01. Source: Behavior Risk Factor Surveillance System 2022–2023. All estimates are adjusted for sampling weight and BRFSS’ complex survey design; confidence intervals are based on standard errors computed using the linearized (or robust) variance estimator.
(DOCX)

S6 Table Full logistic regression models with demographic variables and SDOH variables predicting daily tobacco use, daily cannabis use, and binge drinking behaviors, Adjusted Odds Ratios and 95% Confidence Intervals. *p < 0.05. **p < 0.01.
(DOCX)

S7 Table Variance inflation factors (VIF) for regression models. (DOCX)
