# Supplementary material for: Examining the relationship between social determinants of health with daily tobacco use, binge-drinking, and daily cannabis use
Source: PLoS One. 2026 Mar 18;21(3):e0343677. doi: 10.1371/journal.pone.0343677 (PMC12998838; doi:10.1371/journal.pone.0343677)
Supplement: S1 Table — Source: Behavior Risk Factor Surveillance System 2022–2023. (DOCX) [file pone.0343677.s001.docx]

**S1 Table:** List of States that Elected to Use Cannabis and SDOH Modules by Year

| **Survey Year** | **Cannabis Module** | **SDOH Module** | **Analytic Sample** |
| --- | --- | --- | --- |
| 2022 | Connecticut, Delaware, Guam, Hawaii, Illinois, Indiana, Kansas, Maine, Maryland, Massachusetts, Michigan, Mississippi, Montana, Nebraska, Nevada, New Mexico, North Dakota, Ohio, Oklahoma, Oregon, Virgin Islands, Virginia, Wisconsin, Wyoming (n=24) | Alabama, Alaska, Arizona, California, Connecticut, Delaware, District of Columbia, Florida, Georgia, Idaho, Indiana, Iowa, Kansas, Kentucky, Maine, Maryland, Massachusetts, Michigan, Minnesota, Mississippi, Missouri, Montana, Nebraska, Nevada, New Hampshire, New Jersey, New Mexico, North Carolina, Ohio, Oklahoma, Puerto Rico, Rhode Island, South Carolina, Tennessee, Texas, Utah, Vermont, Virgin Islands, Washington, West Virginia, Wisconsin, Wyoming (n=42) | Connecticut, Delaware, Indiana, Maine, Mississippi, Montana, Nebraska, Nevada, New Mexico, Wisconsin, Wyoming (n=11) |
| 2023 | Connecticut, Delaware, Guam, Illinois, Indiana, Maine, Maryland, Mississippi, Montana, Nebraska, Nevada, New Mexico, Ohio, Oklahoma, Oregon, Vermont, Virgin Islands, Virginia, Wyoming (n=19) | Alabama, Alaska, Arizona, Arkansas, California, Connecticut, Delaware, District of Columbia, Georgia, Hawaii, Idaho, Illinois, Indiana, Iowa, Kansas, Louisiana, Maine, Maryland, Massachusetts, Michigan, Minnesota, Mississippi, Missouri, Montana, Nebraska, Nevada, New Hampshire, New Jersey, New Mexico, New York, North Carolina, North Dakota, Ohio, Oklahoma, Puerto Rico, Rhode Island, South Carolina, Utah, Virginia, West Virginia, Wisconsin (n=41) | Connecticut, Delaware, Illinois, Indiana, Maine, Mississippi, Montana, Nevada, New Mexico, Virginia (n=11) |
| Combined 2022-2023 | | | Connecticut, Delaware, Illinois, Indiana, Maine, Maryland, Mississippi, Montana, Nebraska, Nevada, New Mexico, Virginia, Wisconsin, Wyoming  (n=14) |

Source: Behavior Risk Factor Surveillance System 2022-2023
