# Supplementary material for: Examining the relationship between social determinants of health with daily tobacco use, binge-drinking, and daily cannabis use
Source: PLoS One. 2026 Mar 18;21(3):e0343677. doi: 10.1371/journal.pone.0343677 (PMC12998838; doi:10.1371/journal.pone.0343677)
Supplement: S2 Table — (DOCX) [file pone.0343677.s002.docx]

**S2 Table**: BRFSS Variables Used in Study

| **Study Variable** | **BRFSS Variable Codes** | **BRFSS Questionnaire Items** |
| --- | --- | --- |
| Primary study outcomes | | |
| Daily tobacco use | SMOKDAY2, ECIGNOW2 | Do you now smoke cigarettes every day, some days, or not at all?  Do you now use e-cigarettes, or vaping products every day, some days, or not at all? |
| Daily cannabis use | MARIJAN1 | During the past 30 days, on how many days did you use marijuana or hashish? |
| Binge drinking in past month | DRNK3GE5 | Considering all types of alcoholic beverages, how many times during the past 30 days did you have 5 or more drinks for men or 4 or more drinks for women on an occasion? |
| Social determinants of health variables | | |
| Housing Instability | SDHBILLS | During the last 12 months, was there a time when you were not able to pay your mortgage, rent or utility bills? |
| Food insecurity | SDHFOOD, FOODSTMP | During the past 12 months how often did the food that you bought not last, and you didn’t have money to get more? |
| Transportation problems | SDHTRNSP | During the past 12 months has a lack of reliable transportation kept you from medical appointments, meetings, work, or from getting things needed for daily living? |
| Utility help needs | SDHUTILS | During the last 12 months was there a time when an electric, gas, oil, or water company threatened to shut off services? |
| Interpersonal safety | EMTSUPRT | How often do you get the social and emotional support that you need? |
| Financial strain | MEDCOST1 | Was there a time in the past 12 months when you needed to see a doctor but could not because you could not afford it? |
| Employment | SDHEMPLY | In the past 12 months have you lost employment or had hours reduced?^a^ |
| Family and community support | SDLONELY | How often do you feel lonely? |
| Physical exercise | EXERANY2 | During the past month, other than your regular job, did you participate in any physical activities or exercises such as running, calisthenics, golf, gardening, or walking for exercise? |
| Mental Health | SDHSTRE1 | Stress means a situation in which a person feels tense, restless, nervous or anxious or is unable to sleep at night because their mind is troubled all the time. Within the last 30 days, how often have you felt this kind of stress? |
| Disabilities | DECIDE, DIFFALON | Because of a physical, mental, or emotional condition, do you have serious difficulty concentrating, remembering, or making decisions?  Because of a physical, mental, or emotional condition, do you have difficulty doing errands alone such as visiting a doctor's office or shopping? |
| Healthcare access variables | | |
| Insurance type | PRIMINSI | What is the current source of your primary health insurance? |
| Checkup within the past year | CHECKUP1 | About how long has it been since you last visited a doctor for a routine checkup? |
| Demographic variables | | |
| Age | _AGE_G | Six-level imputed age category [BRFSS calculated variable] |
| Sex | SEXVAR | Sex of respondent |
| Race | RRCLASS3 | How do other people usually classify you in this country? |
| Marital status | MARITAL | Are you: (marital status) |
| Veteran status | VETERAN3 | Have you ever served on active duty in the United States Armed Forces, either in the regular military r in a National Guard or military reserve unit? |
| Education | EDUCA | What is the highest grade or year of school you completed? |
| Employed | EMPLOY1 | Are you currently employed? |
