# Supplementary material for: Examining the relationship between social determinants of health with daily tobacco use, binge-drinking, and daily cannabis use
Source: PLoS One. 2026 Mar 18;21(3):e0343677. doi: 10.1371/journal.pone.0343677 (PMC12998838; doi:10.1371/journal.pone.0343677)
Supplement: S3 Table — Source: Behavior Risk Factor Surveillance System 2022–2023. (DOCX) [file pone.0343677.s003.docx]

**S3 Table.** Number of Observations in the Analytic Sample by State-Year

| **State** | **2022** | **2023** | **Total** |
| --- | --- | --- | --- |
| Connecticut | 9,784 | 9,501 | 19,285 |
| Delaware | 3,987 | 4,282 | 8,269 |
| Illinois | 0 | 5,279 | 5,279 |
| Indiana | 10,466 | 10,993 | 21,459 |
| Maine | 10,646 | 12,255 | 22,901 |
| Maryland | 0 | 5,641 | 5,641 |
| Mississippi | 4,239 | 4,069 | 8,308 |
| Montana | 7,048 | 7,143 | 14,191 |
| Nebraska | 7,473 | 0 | 7,473 |
| Nevada | 3,188 | 2,650 | 5,838 |
| New Mexico | 4,758 | 3,220 | 7,978 |
| Virginia | 0 | 6,981 | 6,981 |
| Wisconsin | 11,276 | 0 | 11,276 |
| Wyoming | 4,142 | 0 | 4,142 |
| TOTAL | 77,007 | 72,014 | 149,021 |

Source: Behavior Risk Factor Surveillance System 2022-2023
