# Supplementary material for: Examining the relationship between social determinants of health with daily tobacco use, binge-drinking, and daily cannabis use
Source: PLoS One. 2026 Mar 18;21(3):e0343677. doi: 10.1371/journal.pone.0343677 (PMC12998838; doi:10.1371/journal.pone.0343677)
Supplement: S4 Table — (DOCX) [file pone.0343677.s004.docx]

**S4 Table:** Adult Use Cannabis Legalization by State-Year

| **State** | **Adult Use** |
| --- | --- |
| Connecticut | 2021 |
| Delaware | 2023 |
| Illinois | 2020 |
| Indiana | NA |
| Maine | 2016 |
| Maryland | 2023 |
| Mississippi | NA |
| Montana | 2021 |
| Nebraska | NA |
| Nevada | 2017 |
| New Mexico | 2021 |
| Virginia | 2021 |
| West Virginia | NA |
| Wisconsin | NA |
| Wyoming | NA |
