# Supplementary material for: Examining the relationship between social determinants of health with daily tobacco use, binge-drinking, and daily cannabis use
Source: PLoS One. 2026 Mar 18;21(3):e0343677. doi: 10.1371/journal.pone.0343677 (PMC12998838; doi:10.1371/journal.pone.0343677)
Supplement: S5 Table — *p < 0.05. **p < 0.01. Source: Behavior Risk Factor Surveillance System 2022–2023. All estimates are adjusted for sampling weight and BRFSS’ complex survey design; confidence intervals are based on standard errors computed using the linearized (or robust) variance estimator. (DOCX) [file pone.0343677.s005.docx]

**S5 Table:** Logistic regression models with only demographic variables predicting daily tobacco use, daily cannabis use, and binge drinking behaviors, Adjusted Odds Ratios and 95% Confidence Intervals

| **Variable** | **Daily Cannabis (n=39,992)** | **Binge Drinking (n=21,363)** | **Daily Tobacco (n=17, 711)** |
| --- | --- | --- | --- |
| **Age (based category: 18-24)** | | | |
| *25-34* | 1.2 | 0.97 | 0.54** |
|  | (0.95 - 1.52) | (0.78 - 1.20) | (0.39 - 0.75) |
| *35-44* | 1.08 | 1.07 | 0.51** |
|  | (0.85 - 1.38) | (0.86 - 1.33) | (0.37 - 0.69) |
| *45-54* | 0.58** | 0.89 | 0.44** |
|  | (0.45 - 0.75) | (0.72 - 1.10) | (0.32 - 0.60) |
| *55-64* | 0.48** | 0.54** | 0.35** |
|  | (0.37 - 0.61) | (0.44 - 0.67) | (0.26 - 0.48) |
| *65+* | 0.17** | 0.30** | 0.15** |
|  | (0.12 - 0.23) | (0.23 - 0.39) | (0.11 - 0.21) |
| **Race (base category: White)** | | | |
| *Black/African American* | 1.24 | 0.69** | 0.79 |
|  | (0.96 - 1.61) | (0.53 - 0.90) | (0.59 - 1.06) |
| *Hispanic/Latino* | 0.47** | 0.87 | 0.51** |
|  | (0.36 - 0.61) | (0.71 - 1.07) | (0.40 - 0.65) |
| *Asian* | 0.26** | 0.86 | 1.68 |
|  | (0.11 - 0.59) | (0.49 - 1.49) | (0.67 - 4.21) |
| *Native American/AIAN* | 0.76 | 1.39 | 0.53** |
|  | (0.54 - 1.07) | (0.92 - 2.10) | (0.37 - 0.77) |
| *Other* | 1.26 | 0.85 | 0.83 |
|  | (0.85 - 1.87) | (0.57 - 1.26) | (0.56 - 1.21) |
| **Sex (based category: female)** | | | |
| *Male* | 1.65** | 1.66** | 0.83** |
|  | (1.44 - 1.90) | (1.49 - 1.84) | (0.73 - 0.93) |
| **Married** | 0.63** | 0.72** | 0.61** |
|  | (0.54 - 0.73) | (0.64 - 0.80) | (0.55 - 0.69) |
| **Veteran** | 1.05 | 0.82* | 1.03 |
|  | (0.84 - 1.31) | (0.69 - 0.97) | (0.87 - 1.22) |
| **Health Insurance Type (based category: Private/Employer)** | | | |
| *Medicare* | 1.80** | 1 | 1.17 |
|  | (1.38 - 2.35) | (0.80 - 1.24) | (0.96 - 1.43) |
| *Medicaid/CHIP* | 2.29** | 0.92 | 1.86** |
|  | (1.81 - 2.90) | (0.73 - 1.16) | (1.51 - 2.29) |
| *Other* | 1.51** | 0.99 | 1.23* |
|  | (1.22 - 1.87) | (0.83 - 1.18) | (1.03 - 1.48) |
| *Uninsured* | 1.11 | 1.14 | 1.44** |
|  | (0.86 - 1.44) | (0.90 - 1.44) | (1.12 - 1.84) |
| **Employed** | 0.96 | 1.25** | 1.03 |
|  | (0.82 - 1.14) | (1.09 - 1.44) | (0.89 - 1.19) |
| **Education (base category: less than high school)** | | | |
| High school | 0.79 | 0.78 | 0.79* |
|  | (0.62 - 1.02) | (0.59 - 1.03) | (0.65 - 0.96) |
| Some college | 0.77 | 0.74* | 0.58** |
|  | (0.60 - 1.00) | (0.56 - 0.97) | (0.47 - 0.71) |
| College graduate | 0.51** | 0.56** | 0.34** |
|  | (0.39 - 0.66) | (0.43 - 0.75) | (0.27 - 0.43) |
| **Resides in a state in which adult use cannabis is legal** | 2.30** | --- | --- |
|  | (2.01 - 2.62) | --- | --- |

*p<0.05

**p<0.01

Source: Behavior Risk Factor Surveillance System 2022-2023

All estimates are adjusted for sampling weight and BRFSS’ complex survey design; confidence intervals are based on standard errors computed using the linearized (or robust) variance estimator.
