# Supplementary material for: Examining the relationship between social determinants of health with daily tobacco use, binge-drinking, and daily cannabis use
Source: PLoS One. 2026 Mar 18;21(3):e0343677. doi: 10.1371/journal.pone.0343677 (PMC12998838; doi:10.1371/journal.pone.0343677)
Supplement: S6 Table — *p < 0.05. **p < 0.01. (DOCX) [file pone.0343677.s006.docx]

**S6 Table.** Full logistic regression models with demographic variables and SDOH variables predicting daily tobacco use, daily cannabis use, and binge drinking behaviors, Adjusted Odds Ratios and 95% Confidence Intervals

| **Variable** | **Daily Cannabis (n=38,332)** | **Binge Drinking (n=20,719)** | **Daily Tobacco (n=16,889)** |
| --- | --- | --- | --- |
| **Age (based category: 18-24)** | | | |
| *25-34* | 1.03 | 0.89 | 0.49** |
|  | (0.81 - 1.32) | (0.71 - 1.11) | (0.35 - 0.68) |
| *35-44* | 0.99 | 1.01 | 0.48** |
|  | (0.77 - 1.28) | (0.81 - 1.26) | (0.35 - 0.67) |
| *45-54* | 0.58** | 0.86 | 0.44** |
|  | (0.45 - 0.76) | (0.69 - 1.07) | (0.32 - 0.61) |
| *55-64* | 0.51** | 0.54** | 0.37** |
|  | (0.40 - 0.66) | (0.43 - 0.67) | (0.27 - 0.51) |
| *65+* | 0.27** | 0.34** | 0.18** |
|  | (0.20 - 0.38) | (0.26 - 0.46) | (0.13 - 0.26) |
| **Race (base category: White)** | | | |
| *Black/African American* | 1.27 | 0.67** | 0.71* |
|  | (0.97 - 1.66) | (0.51 - 0.88) | (0.53 - 0.96) |
| *Hispanic/Latino* | 0.47** | 0.86 | 0.48** |
|  | (0.36 - 0.62) | (0.70 - 1.06) | (0.37 - 0.62) |
| *Asian* | 0.20** | 0.84 | 1.99 |
|  | (0.08 - 0.49) | (0.47 - 1.51) | (0.75 - 5.28) |
| *Native American/AIAN* | 0.79 | 1.3 | 0.53** |
|  | (0.56 - 1.10) | (0.86 - 1.97) | (0.36 - 0.78) |
| *Other* | 1.25 | 0.9 | 0.74 |
|  | (0.81 - 1.91) | (0.60 - 1.36) | (0.49 - 1.11) |
| **Sex (based category: female)** | | | |
| *Male* | 1.81** | 1.75** | 0.88* |
|  | (1.57 - 2.09) | (1.57 - 1.95) | (0.77 - 1.00) |
| **Married** | 0.72** | 0.74** | 0.67** |
|  | (0.61 - 0.85) | (0.66 - 0.82) | (0.59 - 0.75) |
| **Veteran** | 1.06 | 0.83* | 1.1 |
|  | (0.84 - 1.34) | (0.69 - 0.98) | (0.92 - 1.31) |
| **Health Insurance Type (based category: Private/Employer)** | | | |
| *Medicare* | 1.37** | 0.88 | 1.09 |
|  | (1.05 - 1.80) | (0.71 - 1.10) | (0.89 - 1.34) |
| *Medicaid/CHIP* | 1.69*** | 0.82 | 1.58** |
|  | (1.32 - 2.16) | (0.64 - 1.05) | (1.25 - 1.98) |
| *Other* | 1.27** | 0.94 | 1.11 |
|  | (1.01 - 1.59) | (0.78 - 1.14) | (0.92 - 1.34) |
| *Uninsured* | 0.87 | 0.95 | 1.23 |
|  | (0.65 - 1.16) | (0.74 - 1.21) | (0.95 - 1.59) |
| **Employed** | 1.14 | 1.27** | 1.17* |
|  | (0.96 - 1.37) | (1.11 - 1.47) | (1.01 - 1.37) |
| **Education (base category: less than high school)** | | | |
| *High school* | 0.91 | 0.8 | 0.88 |
|  | (0.70 - 1.19) | (0.59 - 1.07) | (0.71 - 1.09) |
| *some college* | 0.91 | 0.76 | 0.64** |
|  | (0.70 - 1.20) | (0.57 - 1.02) | (0.52 - 0.80) |
| *College graduate* | 0.66*** | 0.60** | 0.42** |
|  | (0.49 - 0.88) | (0.45 - 0.81) | (0.33 - 0.53) |
| **Check-up within past year** | 0.82* | 0.84** | 0.77** |
|  | (0.71 - 0.96) | (0.75 - 0.95) | (0.67 - 0.88) |
| **Cannot afford medical care** | 1.40** | 1.31** | 1.01 |
|  | (1.13 - 1.74) | (1.07 - 1.60) | (0.82 - 1.24) |
| **Receives food stamps** | 1.36** | 1.27* | 1.13 |
|  | (1.10 - 1.68) | (1.00 - 1.60) | (0.94 - 1.37) |
| **Food purchased does not last** | 1.23 | 0.92 | 1.09 |
|  | (1.00 - 1.51) | (0.74 - 1.14) | (0.90 - 1.32) |
| **Lack transportation access** | 1.15 | 0.85 | 1.04 |
|  | (0.93 - 1.44) | (0.66 - 1.11) | (0.84 - 1.30) |
| **Housing instability** | 0.88 | 1.1 | 1.19 |
|  | (0.68 - 1.14) | (0.87 - 1.41) | (0.96 - 1.48) |
| **Utility bill needs** | 1.31* | 1.18 | 1.19 |
|  | (1.03 - 1.67) | (0.90 - 1.55) | (0.95 - 1.49) |
| **Has emotional support** | 0.91 | 0.96 | 1.11 |
|  | (0.74 - 1.13) | (0.76 - 1.21) | (0.91 - 1.36) |
| **Job loss within past year** | 1.19 | 0.93 | 1.27** |
|  | (0.99 - 1.45) | (0.78 - 1.12) | (1.06 - 1.52) |
| **Exercise in past 30 days** | 1.16 | 0.87** | 0.72** |
|  | (0.98 - 1.38) | (0.76 - 1.00) | (0.63 - 0.81) |
| **Frequent stress** | 1.43** | 1.27*** | 1.16* |
|  | (1.22 - 1.68) | (1.13 - 1.42) | (1.02 - 1.32) |
| **Difficulty concentrating due to disability** | 1.79** | 1.05 | 1.16 |
|  | (1.49 - 2.16) | (0.88 - 1.26) | (0.98 - 1.38) |
| **Difficulty doing errands alone due to disability** | 1.33* | 0.89 | 1.2 |
|  | (1.07 - 1.65) | (0.69 - 1.13) | (0.98 - 1.47) |
| **Resides in a state in which recreational cannabis is legal** | 2.29** | --- | --- |
|  | (2.00 - 2.63) | --- | --- |

*p<0.05

**p<0.01
