# Supplementary material for: Examining the relationship between social determinants of health with daily tobacco use, binge-drinking, and daily cannabis use
Source: PLoS One. 2026 Mar 18;21(3):e0343677. doi: 10.1371/journal.pone.0343677 (PMC12998838; doi:10.1371/journal.pone.0343677)
Supplement: S7 Table — (DOCX) [file pone.0343677.s007.docx]

**S7 Table.** Variance inflation factors (VIF) for regression models

| **Variable** | **Outcome: Daily Cannabis** | **Outcome: Binge Drinking** | **Outcome: Daily Tobacco** |
| --- | --- | --- | --- |
| **Age** | | | |
| *18-24* | *Base* | *Base* | *Base* |
| *25-34* | 2.86 | 3.08 | 4.7 |
| *35-44* | 3.56 | 3.77 | 7.48 |
| *45-54* | 4 | 4.11 | 8.18 |
| *55-64* | 4.88 | 4.82 | 10.57 |
| *65+* | 8.57 | 8.62 | 17.44 |
| **Race** | | | |
| *White* | *Base* | *Base* | *Base* |
| *Black/African American* | 1.03 | 1.03 | 1.03 |
| *Hispanic/Latino* | 1.11 | 1.08 | 1.07 |
| *Asian* | 1.01 | 1.01 | 1.01 |
| *Native American/AIAN* | 1.04 | 1.03 | 1.04 |
| *Other* | 1.01 | 1.01 | 1.01 |
| **Sex** | | | |
| *Female* | *Base* | *Base* | *Base* |
| *Male* | 1.21 | 1.18 | 1.25 |
| **Married** | 1.17 | 1.17 | 1.25 |
| **Veteran** | 1.26 | 1.25 | 1.14 |
| **Health Insurance Type** | | | |
| *Private/Employer* | *Base* | *Base* | *Base* |
| *Medicare* | 2.81 | 3.1 | 2.7 |
| *Medicaid/CHIP* | 1.37 | 1.33 | 1.47 |
| *Other* | 1.31 | 1.26 | 1.43 |
| *Uninsured* | 1.21 | 1.18 | 1.25 |
| **Employed** | 1.81 | 1.86 | 1.84 |
| **Education** | | | |
| *Less than High School* | *Base* | *Base* | *Base* |
| *High school* | 5.3 | 7.45 | 4.42 |
| *some college* | 5.82 | 9.06 | 4.59 |
| *College graduate* | 6.93 | 11.18 | 4.78 |
| **Check-up within past year** | 1.13 | 1.13 | 1.15 |
| **Cannot afford medical care** | 1.24 | 1.24 | 1.26 |
| **Receives food stamps** | 1.4 | 1.38 | 1.47 |
| **Food purchased does not last** | 1.41 | 1.38 | 1.45 |
| **Lack transportation access** | 1.28 | 1.28 | 1.33 |
| **Housing instability** | 1.71 | 1.68 | 1.79 |
| **Utility bill needs** | 1.42 | 1.4 | 1.48 |
| **Has emotional support** | 1.1 | 1.09 | 1.11 |
| **Job loss within past year** | 1.13 | 1.15 | 1.15 |
| **Exercise in past 30 days** | 1.1 | 1.07 | 1.1 |
| **Frequent stress** | 1.24 | 1.22 | 1.3 |
| **Difficulty concentrating due to disability** | 1.25 | 1.26 | 1.3 |
| **Difficulty doing errands alone due to disability** | 1.21 | 1.18 | 1.24 |
